# Identification of gene clusters differentially expressed during the cellular injury responses (CIR) to cisplatin

**DOI:** 10.1054/bjoc.2001.2080

**Published:** 2001-10

**Authors:** A Johnsson, P Byrne, R de Bruin, D Weiner, J Wong, G Los

**Affiliations:** UCSD Cancer Center, University of California, San Diego, La Jolla, CA, 92037-0058, USA

**Keywords:** cisplatin, suppression subtractive hybridization, high throughput screening, gene expression

## Abstract

The goal of this study was to identify changes in mRNA levels in tumour cells after a toxic exposure to cisplatin (IC_99_dose). Using suppression-subtractive hybridization (SSH) 2 cDNA libraries were created, an UP library (202 cDNA fragments) and a DOWN library (153 cDNA fragments). Using reversed Northern hybridization 16 and 30 fragments were truly differentially expressed in the UP and DOWN libraries, respectively. Most prominent in the UP library were the mitochondrial and injury response clusters and in the DOWN library the cytoskeletal, protein synthesis and signalling clusters. These distinct clusters potentially represent an expression profile of the cisplatin-induced cellular injury response. © 2001 Cancer Research Campaign  http://www.bjcancer.com


British Journal of Cancer (2001) 85(8), 1206–1210
© 2001 Cancer Research Campaign
doi: 10.1054/ bjoc.2001.2080, available online at http://www.idealibrary.com on IDEAL

http://www.bjcancer.com

Short Communication

### Identification of gene clusters differentially expressed during the cellular injury responses (CIR) to cisplatin

**A Johnsson, P Byrne, R de Bruin, D Weiner, J Wong and G Los**

UCSD Cancer Center, University of California, San Diego, La Jolla, CA 92037-0058, USA

**Summary** The goal of this study was to identify changes in mRNA levels in tumour cells after a toxic exposure to cisplatin ($IC_{99}$ dose). Using suppression-subtractive hybridization (SSH) 2 cDNA libraries were created, an UP library (202 cDNA fragments) and a DOWN library (153 cDNA fragments). Using reversed Northern hybridization 16 and 30 fragments were truly differentially expressed in the UP and DOWN libraries, respectively. Most prominent in the UP library were the mitochondrial and injury response clusters and in the DOWN library the cytoskeletal, protein synthesis and signalling clusters. These distinct clusters potentially represent an expression profile of the cisplatin-induced cellular injury response. © 2001 Cancer Research Campaign http://www.bjcancer.com

**Keywords**: cisplatin; suppression subtractive hybridization; high throughput screening, gene expression

Cisplatin (cDDP) is a commonly used anticancer agent for treatment of a variety of different malignancies. It is thought to act by forming adducts with cellular DNA, which leads to inhibition of DNA replication (Pinto and Lippard, 1985) RNA transcription (Corda et al, 1991), as well as activation of the cellular injury response (CIR) (Howell et al, 1996). The latter requires a system for detection of DNA damage (Toney et al, 1989), assessment of the extent of the damage (Eastman, 1990) and finally a system to initiate either repair or apoptosis (Eastman, 1990). Each phase in this process from damage recognition to repair or apoptosis needs the participation of a large number of gene products. Therefore, it seems reasonable to assume that the cDDP-induced CIR can be characterized by the sum of changes in expression level of many specific genes (Howell et al, 1996). In view of the fact that little is known about the specific pathways activated by cDDP-induced CIR, the main goal of this study is to identify genes whose expression level is altered after exposure to cDDP and to classify these genes according to their function. For this purpose we combined the suppression subtractive hybridization technique (Diatchenko et al, 1996) with a custom array technology, which has been recently reported to be productive in identifying differentially expressed genes in other model systems as well (Kuang et al, 1998; Johnsson et al, 2000).

### MATERIAL AND METHODS

### Cells

UMSCC10b human squamous cell carcinoma cells (Grenman et al, 1991) were cultured in RPMI 1640 (Irvine Scientific, Santa Ana, CA) supplemented with 2 mM L-glutamine, 100 units $ml^{-1}$ of penicillin G, 100 mg $ml^{-1}$ of streptomycin sulfate and 10% fetal bovine serum (Gibco BRL, Grand Island, NY).

*Received 11 April 2001*
*Revised 17 July 2001*
*Accepted 19 July 2001*

*Correspondence to:* G Los

### mRNA extraction

The mRNA used for library construction was isolated from 80% confluent cells by acid guanidium phenol-chloroform extraction followed by isolation of poly(A)+ mRNA using the Oligotex mRNA Midi Kit (Qiagen Inc, Chatsworth, CA). The mRNA used to make cDNA-probes directly from tumour cells was isolated by using the mRNA Direct Kit (Qiagen).

### Suppression subtractive hybridization

SSH was performed using the ClonTech PCR-select cDNA Subtraction kit (Clontech Laboratories Inc, Palo Alto, CA) according to the manufacturer’s instructions and as described in detail by Johnsson et al (2000).

### Preparation of membrane arrays

PCR amplified cDNA fragment from the bacterial clones were spotted onto Magna Graph nylon membranes (Micron Separation Inc, Westborough, MA). Each membrane consisted of a maximum of 108 spots. Water, β-actin, adaptor sequences corresponding to the nested primers and serial dilutions of the whole population of cDNA fragments recovered from forward or reverse SSH steps were included as internal controls (Johnsson et al, 2000).

### Membrane hybridizations

Two types of hybridization probes were used in this study. The first was a PCR-amplified probe, containing cDNA fragments recovered from either the forward or reversed SSH step and which putatively contained only cDNA fragments corresponding to differentially expressed mRNAs (Diatchenko et al, 1996; Johnsson et al, 2000). The second probe was used to perform reversed Northern hybridizations. It consisted of cDNA from cDDP-treated and -untreated UMSCC10b cells, prepared by reverse transcription of total cellular mRNA. All probes were labelled with $^{32}P$ by utilizing the Multiprime Labeling Kit (Amersham Life Science, Arlington Heights, IL), with 20 ng of cDNA per probe (specific activity ranged from $5 \times 10^7$ to $8 \times 10^8$ cpm $\mu g^{-1}$ DNA). Using these probes (5 ng $ml^{-1}$) membranes were hybridized for 16 h at 68˚C. Hybridizations with subtracted SSH-derived PCR-amplified probes (screening) were performed in triplicate and hybridization with cDNA non-amplified cDNA probes (reversed northerns) were performed in duplicate, triplicate or quadruplet. Analysis of the hybridization was performed with an imaging system from Bio-Rad Laboratories, Hercules, CA and the data were analysed with the PC-based Molecular Analyst Software.

### Sequencing and identification of identified fragments

Plasmids containing cDNA fragments that were differentially expressed were sequenced using either primers homologous to the M13 reversed priming site of the plasmid, or nested primers targeted to adaptors 1 or 2R. The sequencing was performed with a 373 XL Automated DNA Sequencer (Perkin-Elmer/Applied Biosystems, Norwalk, CT) at the UCSD Core Facility.

### RESULTS AND DISCUSSION

### Library construction and differential screening

SSH was used to create a population of cDNA fragments corresponding to mRNAs whose levels were either increased (the UP library) or decreased (the DOWN library, created by the reversed subtraction using the non-treated cells as tester) in the UMSCC10b cells exposed to an $IC_{99}$ concentration (50 μM) of cDDP relative to the untreated UMSCC10b cells. Figure 1 presents a flow diagram of the yield from each step of the isolation procedure.

The PCR products generated from the bacterial inserts were screened for differential expression with PCR-amplified SSH probes on membrane arrays (Diatchenko et al, 1996; Johnsson et al, 2000). Array elements demonstrating >5-fold differences in abundance in the UP and DOWN subtracted libaries in at least 1 of 3 repeat hybridizations were selected for further investigation. Based on this criterion, 26 (13%) and 44 (29%) fragments were identified as being differentially expressed in the UP and DOWN library, respectively. Although modest, these percentages are of the same order of magnitude as demonstrated in a recent study from this laboratory (Johnsson et al, 2000).

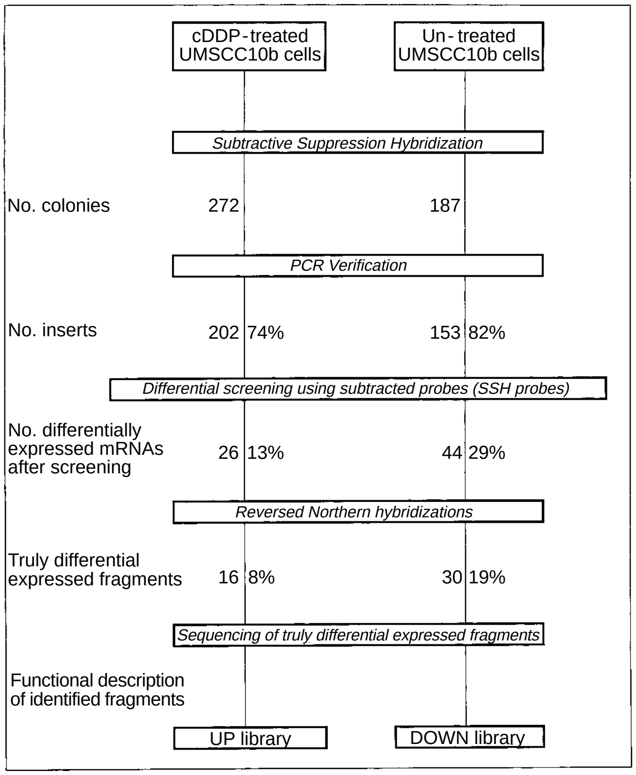

**Figure 1** Flow diagram showing the number of cDNA fragments processed for the UP and DOWN libraries

### Magnitude of differential expression determined by reversed Northern blot analysis

The magnitude of the difference in abundance between the mRNA levels in the cDDP-exposed and non-exposed UMSCC10b cells were examined further by reversed Northern blotting. 70 cDNAs present in the UP (26) and DOWN (44) libraries were spotted onto membrane arrays and hybridized with cDNA probes prepared from mRNA of cells exposed and non-exposed to cDDP ($IC_{99}$). Hybridizations were repeated 2 to 4 times and the mean level of differential expression for each element in the array was calculated as the ratio between cDDP-exposed and -non-exposed cells. Of the 26 cDNAs present in the UP library, 16 (62%) corresponded to mRNAs that demonstrated at least a 1.6-fold difference in level. Among these 16 cDNAs 4 showed at least a 4-fold difference and 1 at least a 10-fold difference (Table 1). Of the 44 cDNAs in the DOWN library, 30 (68%) demonstrated at least a 1.6-fold difference in expression. Among these 6 cDNAs showed at least a 4-fold difference. None had a 10-fold difference (Table 2). The finding that the majority of mRNAs show little change, and a progressively smaller fraction shows incrementally larger changes is consistent with results obtained in other systems where either isogenic cells growing under different conditions have been compared (Zhang et al, 1997) or in a steady state condition where tumour cells resistant to cDDP were compared to their sensitive variant (Johnsson et al, 2000).

Validation of the magnitude of expressing was obtained using 1 or 2 fragments in each library present multiple times. In the DOWN library β-actin and α-tubulin were identified 4 and 3 times, respectively. The mean expression of their expression ratios was $3.4 \pm 1.5$ and $2.9 \pm 0.9$, respectively. In the UP library MDM2 was present in 4 different bacterial clones, resulting in expression levels $1.9 \pm 0.1$. Overall, the mean variance is small and confirms previous observations of biological variation between samples (Zhang et al, 1997; Johnsson et al, 2000).

### Identification of cDNA fragments

The cDNA fragments corresponding to the 16 mRNAs in the UP library and 30 mRNAs in the DOWN library that demonstrated at least a 1.6-fold increase or decrease in expression were sequenced. 43 (92%) of these were identifiable as segments of cDNAs contained in GenBank, 1 (2%) was an EST and 3 (6%) were unknown. Some genes were identified more than once. mRNA encoding mitochondrial genes and MDM2 were identified multiple times in the UP library, and β-actin, and α-tubulin were found more than once in the DOWN library. In the UP library 2 clusters of genes can be identified (Table 1). The first cluster consists of mitochondrial related genes, the 16S rRNAs and BC200. The human mtDNA encoding rRNAs (rRNAs 12 S and 16S rRNA) are involved in the synthesis of several subunits of

© 2001 Cancer Research Campaign

**Table 1** Truly differentially expressed fragments present in the UP library

| Clone number | Fragment identity | GenBank identity | GenBank accession # | Fold increase in expression | Function |
|---|---|---|---|---|---|
| | | | | | ***Ribosomal products*** |
| 326 | mitochondrion (2851–3089) | HUMMTA | *D38112* | 13.7 | mtDNA coding for 16S rRNA |
| 230b | mitochondrion (2849–3124) | HUMMTA | *D38112* | 6.2 | mtDNA coding for 16S rRNA |
| 119b | mitochondrion (2332–2544) | HUMMTA | *D38112* | 3.9 | mtDNA coding for 16S rRNA |
| 313 | mitochondrion (2282–2629) | HUMMTA | *D38112* | 2.1 | mtDNA coding for 16S rRNA |
| 46a | BC200 | HSBC200RNA | *U01305* | 2.1 | Associated with 5S rRNA |
| | | | | | ***Genes involved in the CIR*** |
| 83b | hMSH6 | HSU54777 | *U54777* | 3.5 | DNA damage recognition and repair |
| 203b | HSP90 | HSHSP90R | *X15183* | 1.8 | Early response gene, heat shock protein |
| 339 | MDM2 | F144014S16 | *AF144029* | 2.0 | Negative feedback for p53 |
| 336 | MDM2 | F144014S16 | *AF144029* | 1.9 | Negative feedback for p53 |
| 280 | MDM2 | F144014S16 | *AF144029* | 1.9 | Negative feedback for p53 |
| 247 | MDM2 | F144014S16 | *AF144029* | 1.7 | Negative feedback for p53 |
| | | | | | ***Function unknown*** |
| 165b | DDX1 (dead box protein) | HSCL1042 | *X70649* | 2.4 | Translation, RNA splicing and RNA stability |
| 299 | NICE-5 protein | NM_017582 | *NM_017582* | 1.8 | Part of human epidermal differentiation complex |
| 65b | Human clone JkA7 mRNA | HSU38436 | *U38436* | 1.7 | unknown |
| 94b | KIAA0017 | HUMRSC399 | *D13642* | 4.1 | unknown |
| 242 | unknown | | | 1.6 | unknown |

among others the cytochrome C oxidase complex, ATPases and the NADH reductase complex (Darnell et al, 1990), all known to be potentially associated with the apoptotic process. Little is known about the function of BC200, however, it has been shown that BC200 is associated with polymerase III transcripts, which by itself makes a variety of small stable RNAs including the small 5S RNA of the ribosome (Kremerskothen et al, 1998).

A second cluster consists of genes directly involved in the cellular injury response (CIR) such as hMSH6, HSP90 and MDM2. The hMSH6 is a part of the DNA mismatch repair system and forms a complex with hMSH2 that recognizes and initiates repair of DNA damage (Lage et al, 1999). In analogy of its role in DNA mismatch repair, it has been shown that hMSH6 may also play a role in the recognition of cDDP-DNA adducts (Fink et al, 1996) and therefore may play a role in the initiation of the CIR. Another important player in the CIR is HSP90 (Itoh and Tashima, 1991). HSP90 gets induced by cDDP under conditions of severe damage as was demonstrated in mice kidney cells with cDDP-induced acute renal failure and degenerative changes in epithelial cells (Satoh et al, 1994). Part of the function of HSP90 is to chaperone the folding of proteins and by doing so play a role in regulating signal transduction pathways that control cell growth and survival. A third member of this CIR cluster is the *MDM2* gene, an important player in the p53 pathway through the formation of a MDM2–p53 auto-regulatory feedback loop (Oliner et al, 1993). For several other identified cDNA fragments the role in the CIR is less clear. The dead box protein gene *DDX1* might be involved in translation, RNA splicing and RNA stability, while the function of NICE-5, JkA7, KIAA0017 and cDNA fragment are unknown.

In the DOWN library 3 functional gene clusters could be identified, involving cell structure, signalling and protein translation. The first cluster contains genes such as α-tubulin, β-actin, cytokeratin, actin-binding protein, keratin-related protein, cytokeratin 13, and thymosin β4. The changes in expression level of a number of cytoskeletal components suggest a reorganization of the microfilament system in response to the cDDP-induced injury which has also been observed during the process of cell death (Brancolini et al, 1997). Cells retract from adhesion substrate, sever the contacts with neighbouring cells, and actin filaments will be concentrated in the perinuclear area in a ring-like organization, resulting in among others, the down-regulation of the actin-binding protein and thymosin $\beta_4$, an actin-sequestering protein (Huff et al, 1999) and the rupture of α-tubulin during the apoptotic process (Kato, 1999). The function of the Wilm's tumour-related protein in human is unknown, however, its yeast homologue GRC5, seems to be an essential yeast gene for the establishment of a proper cytoskeletal structure (Koller et al, 1996). Summarizing, the parallel down-regulation of the expression level of so many cytoskeletal-related genes strongly suggests a well-orchestrated process dismantling the structure of the cell.

A second cluster contains a set of genes involved in various signalling pathways. Transketolase and the thyroid hormone-binding protein are both part of the metabolic energy pathways in the cell. Transkelotase is part of the pentose phosphate pathway (Pontremoli and Grazi 1968) and the thyroid hormone-binding protein gene encodes a monomer of pyruvate kinase, which can lead to decreased pyruvate kinase activity (Dieudonne et al, 1999). Two other genes, *ferritin H chain* and transcription factor *HSF4b*, are involved in pathways activated by oxidative stress or damage. Ferritin synthesis plays a major role in the prevention of cellular damage (Balla et al, 1992) while the transcription factor *HSF4b* acts as a transcriptional activator of the heat-shock proteins (Tanabe et al, 1999). This cluster also contains the interferon-inducible gene family *1-8U*. This gene is induced by both type 1 (α and β) and type II (γ) IFN. The function of IFN-inducible gene *1-8U* remains, however, unclear. Taken this all together, it is clear that the down-regulation of genes present in this cluster negatively affects the cells' energy metabolism and the ability to react on stress.

A third cluster in the DOWN library indicates that the protein synthesis is reduced at several levels. First the expression levels of elongation factors 1-alpha and 1-gamma, which are known to

 *© 2001 Cancer Research Campaign*

**Table 2** Truly differentially expressed fragments present in the DOWN library

| Clone number | Fragment identity | GenBank identity | GenBank accession # | Fold increase in expression | Function |
|---|---|---|---|---|---|
| | | | | | ***Signaling*** |
| 119 | PABPCI | HUMPOLYAB | *NM 002568* | 6.7 | mRNA stability |
| 159 | transcription factor HSF4b | AB029348 | *AB029348* | 2.3 | transcriptional activator of HSP |
| 44 | Ferritin H chain | HUMMFERH | *M11146* | 2.3 | cellular damage prevention |
| 196 | Interferon-inducible gene | HS18U | *X57352* | 1.6 | part of the IFN pathway |
| 126 | Transketolase | HSU55017 | *U55017* | 2.3 | pentose phosphate pathway |
| 38 | Thyroid hormone binding protein | HUMTCBA | *M26252* | 8.7 | affect pyruvate kinase activity |
| | | | | | ***Protein Synthesis*** |
| 66 | Elongation factor 1-$\alpha$ | HUMEF1A | *J04617* | 6.9 | } protein translation |
| 127 | Elongation factor 1-$\gamma$ | HSEF1GMR | *Z11531* | 2.0 | |
| 116 | Acidic ribosomal phosphoprotein | HUMPPARPO | *M17885* | 2.6 | } |
| 25 | Ribosomal protein S29 | HSU14973 | *U14973* | 2.5 | ribosome regulation |
| 195 | Ribosomal protein L41 | HSRPL41 | *Z12962* | 1.7 | |
| 199 | Proteasome subunit HC3 | HUMPSC3 | *D00760* | 2.0 | protein breakdown |
| | | | | | ***Cytoskeletal Organization*** |
| 100 | $\beta$-actin | HSAC07 | *X00351* | 5.6 | } |
| 109 | $\beta$-actin | HSAC07 | *X00351* | 2.8 | |
| 53 | $\beta$-actin | HSAC07 | *X00351* | 2.6 | |
| 192 | $\beta$-actin | HSAC07 | *X00351* | 2.5 | cytosketal organization |
| 35 | Actin-binding protein | HSABP280 | *X53416* | 2.7 | protein synthesis |
| 9 | Keratin-related protein | HSKERELP | *X62571* | 4.4 | cytoskeleton dynamics |
| 154 | $\alpha$-tubulin | HUMTUBAK | *K00558* | 4.0 | differentiation |
| 139 | $\alpha$-tubulin | HUMTUBAK | *K00558* | 2.7 | |
| 189 | $\alpha$-tubulin | HUMTUBAK | *K00558* | 2.1 | |
| 153 | Wilm's tumor related protein | HUMQM | *M64241* | 2.5 | |
| 6 | Cytokeratin 13 | HSCYTK | *X52426* | 3.8 | |
| 15 | Thymosin $\beta_4$ | HUMTHYB4 | *M17733* | 3.5 | actin sequestering protein |
| | | | | | ***Function unknown*** |
| 22 | Cloride ion current inducer | HSU53454 | *U53454* | 2.7 | unknown |
| 47 | Insulinoma gene | HUMIDB | *J02984* | 2.5 | possible DNA-binding protein |
| 122 | Retinal pigment epithelium | HUMRETP1GB | *L07393* | 2.5 | unknown |
| 36 | Unknown | | *NM_003917* | 2.4 | unknown |
| 106 | cDNA FLJ11896 fis | AK021958 | *AK021958* | 2.3 | unknown |
| 81 | Unknown | | | 1.6 | unknown |

mediate the binding of aminoacyl-tRNAs to acceptor sites of ribosomes during protein synthesis (Duttaroy et al, 1998), are down-regulated. Second, exposure to cDDP negatively affects the synthesis as well as the number of active ribosomes as demonstrated by the decrease in level of expression of several ribosomal RNAs (Jordan and Carmo-Fonseca, 1998). Thirdly, the expression level of the proteasome subunit *HC3*, a gene involved in protein breakdown is reduced as well and may serve as a feedback reaction on the reduced protein production (Tiao et al, 1997). For the remaining genes the function is either unknown or not well understood yet.

In summary, the present study used a PCR-based subtraction strategy to provide an analysis of the cellular injury response to cDDP. The cytotoxic response to cDDP in UMSCC10b cells involved the induction as well as the repression of genes. The majority of these genes identified as differentially expressed could be grouped in clusters based on their functional description. These clusters included genes involved in energy production, response to damage, organization of the cytoskeleton, protein synthesis and signalling transduction, providing a map for an expression profile associated with the injury response to cDDP, underlining the feasibility of using a molecular profile or fingerprint of anticancer drugs in tumours as a prognostic tool.

### ACKNOWLEDGEMENTS

This study was supported in part by grants RO1 CA77618 from the National Cancer Institute and RPG-99-159-01 from the American Cancer Society and was conducted in part by the Clayton Foundation for Research-California Division. Dr Los is a Clayton Foundation Researcher.

### REFERENCES

Balla G, Jacob HS, Balla J, Rosenberg M, Nath K, Apple F, Eaton JW and Vercellotti GM (1992) Ferritin: a cytoprotective antioxidant strategem of endothelium. *J Biol Chem* **267**: 18148–18153

Brancolini C, Lazarevic D, Rodriguez J and Schneider C (1997) Dismantling cell–cell contacts during apoptosis is coupled to a caspase-dependent proteolytic cleavage of beta-catenin. *J Cell Biol* **139**: 759–771

Corda Y, Job C, Anin MF, Leng M and Job D (1991) Transcription by eucaryotic and procaryotic RNA polymerases of DNA modified at a d(GG) or a d(AG) site by the antitumor drug cis-diamminedichloroplatinum(II). *Biochemistry* **30**: 222–230

Darnell J, Lodish H and Baltimore D (1990) Organelle biogenesis: the nucleus, chloroplast and mitochondria. In *Molecular cell biology*. J Darnell, HLD Baltimore (ed) pp. 686–699. Scientific American Books, Inc: New York, NY

Diatchenko L, Lau YF, Campbell AP, Chenchik A, Moqadam F, Huang B, Lukyanov S, Lukyanov K, Gurskaya N, Sverdlov ED and Siebert PD (1996) Suppression

*© 2001 Cancer Research Campaign*

subtractive hybridization: a method for generating differentially regulated or tissue-specific cDNA probes and libraries. *Proc Natl Acad Sci USA* **93**: 6025–6030

Dieudonne SC, Kerr JM, Xu T, Sommer B, DeRubeis AR, Kuznetsov SA, Kim IS, Gehron Robey P and Young MF (1999) Differential display of human marrow stromal cells reveals unique mRNA expression patterns in response to dexamethasone. *J Cell Biochem* **76**: 231–243

Duttaroy A, Bourbeau D, Wang XL and Wang E (1998) Apoptosis rate can be accelerated or decelerated by overexpression or reduction of the level of elongation factor-1 alpha. *Exp Cell Res* **238**: 168–176

Eastman A (1990) Activation of programmed cell death by anticancer agents: cisplatin as a model system. *Cancer Cells* **2**: 275–280

Fink D, Nebel S, Aebi S, Zheng H, Cenni B, Nehme A, Christen RD and Howell SB (1996) The role of DNA mismatch repair in platinum drug resistance. *Cancer Res* **56**: 4881–4886

Grenman R, Carey TE, McClatchey KD, Wagner JG, Pekkola-Heino K, Schwartz DR, Wolf GT, Lacivita LP, Ho L, Baker SR et al (1991) In vitro radiation resistance among cell lines established from patients with squamous cell carcinoma of the head and neck. *Cancer* **67**: 2741–2747

Howell SB, Gately DP, Christen RD and Los G (1996) The cisplatin-induced cellular injury response. Proceedings of the ISPCC, in “Platinum and other Metal coordination compounds in Cancer Chemotherapy 2”,. *Plenum Press, New York* 269–282

Huff T, Ballweber E, Humeny A, Bonk T, Becker C, Muller CS, Mannherz HG and Hannappel E (1999) Thymosin beta(4) serves as a glutaminyl substrate of transglutaminase. Labeling with fluorescent dansylcadaverine does not abolish interaction with G-actin. *FEBS Lett* **464**: 14–20

Itoh H and Tashima Y (1991) The stress (heat shock) proteins. *Int J Biochem* **23**: 1185–1191

Johnsson A, Zeelenberg I, Min Y, Hilinski J, Berry C, Howell SB and Los G (2000) Identification of genes differentially expressed in association with acquired cisplatin resistance [In Process Citation]. *Br J Cancer* **83**: 1047–1054

Jordan P and Carmo-Fonseca M (1998) Cisplatin inhibits synthesis of ribosomal RNA in vivo. *Nucleic Acids Res* **26**: 2831–2836

Kato MV (1999) The mechanisms of death of an erythroleukemic cell line by p53: involvement of the microtubule and mitochondria. *Leuk Lymphoma* **33**: 181–186

Koller HT, Klade T, Ellinger A and Breitenbach M (1996) The yeast growth control gene GRC5 is highly homologous to the mammalian putative tumor suppressor gene QM. *Yeast* **12**: 53–65

Kremerskothen J, Nettermann M, op de Bekke A, Bachmann M and Brosisus J (1998) Identification of human autoantigen La/SS-B as BC1/BC200 RNA-binding protein. *DNA Cell Biol* **17**: 751–759

Kuang WW, Thompson DA, Hoch RV and Weigel RJ (1998) Differential screening and suppression subtractive hybridization identified genes differentially expressed in an estrogen receptor-positive breast carcinoma cell line. *Nucleic Acids Res* **26**: 1116–1123

Lage H, Christmann M, Kern MA, Dietel M, Pick M, Kaina B and Schadendorf D (1999) Expression of DNA repair proteins hMSH2, hMSH6, hMLH1, O6-methylguanine-DNA methyltransferase and N-methylpurine-DNA glycosylase in melanoma cells with acquired drug resistance. *Int J Cancer* **80**: 744–750

Oliner JD, Pietenpol JA, Thiagalingam S, Gyuris J, Kinzler KW and Vogelstein B (1993) Oncoprotein MDM2 conceals the activation domain of tumour suppressor p53. *Nature* **362**: 857–860

Pinto AL and Lippard SJ (1985) Sequence-dependent termination of in vitro DNA synthesis by cis- and trans-diamminedichloroplatinum (II). *Proc Natl Acad Sci USA* **82**: 4616–4619

Pontremoli S and Grazi E (1968) Gluconeogenesis. In *Carbohydrate Metabolism and its disorders*, Dickens F, Randle PJ and Whelan WJ (ed), Vol. 1. pp. 259–295. Academic Press

Satoh K, Wakui H, Komatsuda A, Nakamoto Y, Miura AB, Itoh H and Tashima Y (1994) Induction and altered localization of 90-kDa heat-shock protein in rat kidneys with cisplatin-induced acute renal failure. *Ren Fail* **16**: 313–323

Tanabe M, Sasai N, Nagata K, Liu XD, Liu PC, Thiele DJ and Nakai A (1999) The mammalian HSF4 gene generates both an activator and a repressor of heat shock genes by alternative splicing. *J Biol Chem* **274**: 27845–27856

Tiao G, Hobler S, Wang JJ, Meyer TA, Luchette FA, Fischer JE and Hasselgren PO (1997) Sepsis is associated with increased mRNAs of the ubiquitin-proteasome proteolytic pathway in human skeletal muscle. *J Clin Invest* **99**: 163–168

Toney JH, Donahue BA, Kellett PJ, Bruhn SL, Essigmann JM and Lippard SJ (1989) Isolation of cDNAs encoding a human protein that binds selectively to DNA modified by the anticancer drug cis-diamminedichloroplatinum(II) [published erratum appears in Proc Natl Acad Sci USA 1990 Feb; 87(4): 1625]. *Proc Natl Acad Sci USA* **86**: 8328–8332

Zhang L, Zhou W, Velculescu VE, Kern SE, Hruban RH, Hamilton SR, Vogelstein B and Kinzler KW (1997) Gene expression profiles in normal and cancer cells. *Science* **276**: 1268–1272

*© 2001 Cancer Research Campaign*

